# Benefits of ART simplification on adherence, clinical and economic outcomes

**DOI:** 10.7448/IAS.15.6.18064

**Published:** 2012-11-11

**Authors:** J Vera, F Aragão, M Guimaraes, I Vaz Pinto

**Affiliations:** 1 Centro Hospitalar de Cascais, Cascais, Portugal; 2 Escola Nacional de Saúde Pública and Associação SER+, Lisbon, Portugal

## Abstract

**Purpose of the study:**

Fixed-dose combinations (FDCs) and single-tablet regimens (STRs) may reduce complete non-adherence (CNA) but also have the potential additional benefit of avoiding partial non-adherence (PNA) (some but not all drugs taken). We test this hypothesis and then estimate the impact of PNA-free regimen (STRs) on clinical and economic outcomes.

**Methods:**

The unit analysis was a person-regimen of 2 NRTI+(NNRTI, PI/r) lasting ≥90 days. Adherence was measured by the proportion of days covered using pharmacy refill data. The Wilcoxon rank-sum test was used to compare CNA, PNA and total non-adherence (TNA) in the following groups: STR vs its components (EFV+TDF/FTC) and non-FDC vs FDC vs STR. The level of adherence per group and the impact of PNA and CNA on virological failure (VF) were estimated using multivariate regression panel data models (MVRPDM). Control variables included age, gender, hepatitis co-infection, cumulative comorbidity score, CD4, viral load, regimen duration and calendar year. MVRPDM we also used to access the impact of PNA-free regimens on the probability of at least one hospitalization and on annualized hospitalization+ART costs. The analysis was performed in Stata 11^®^.

**Summary of results:**

The retrospective analysis was performed on 2,449 person-regimens from a cohort of 1,435 HIV-infected individuals followed in one HIV unit in Portugal, between 2001 and 2011. Median age was 38 years old and median regimen duration was 1.9 years. CNA was higher (23%) in the non-FDC (p<0.001 vs other groups) and similar in FDC vs STR (15% vs 11%, p=0.951). PNA was higher in non-FDC than FDC (4.2% vs 3.4%; p=0.020) and obviously null with STR (p<0.001 vs other groups). In the subgroup analysis of EFV+FTC/TDF vs STR, TNA was 16% and 11% (p=0.0025). In MVRPDM, STRs are estimated to result in a decrease of 11% (p<0.001) and 5.3% (p<0.001) in TNA relatively to non-FDC and FDC, respectively. Both CNA and PNA were found to be predictors of VF (p<0.001 and p=0.020, respectively). The odds ratio of hospitalization with STRs vs other regimens was 0.33 (p=0.019). STRs were associated with lower sum of ART+hospitalization annualized costs (−1,330€; p<0.001) when compared to other regimens.

**Conclusions:**

This study suggests that FDC and STRs are of value in avoiding PNA and TNA, thereby reducing the probability of virological failure, and that PNA-free regimens are associated with clinical and economic benefits.

